# Editorial on the Special Issue “Obstructive Sleep Apnea (OSA)”

**DOI:** 10.3390/life14060669

**Published:** 2024-05-23

**Authors:** Konstantinos Chaidas

**Affiliations:** 1 Ear, Nose, and Throat Department, University Hospital of Alexandroupolis, School of Medicine, Democritus University of Thrace, 68100 Alexandroupolis, Greece; konchaidas@gmail.com; 2 Ear, Nose, and Throat Department, Imperial College Healthcare NHS Trust, London W2 1NY, UK

Obstructive sleep apnea (OSA) is the most common form of sleep-disordered breathing and is characterized by recurrent episodes of complete or partial upper airway obstruction during sleep, resulting in oxygen desaturation, autonomic dysfunction and sleep fragmentation. It can affect both children and adults, and the main clinical symptoms include loud snoring, noticeable apneas and breathing difficulties during sleep. Overnight polysomnography is the gold-standard method for diagnosing OSA. Although OSA is common, it is a frequently unrecognized cause of serious disabilities that has serious health and social consequences. If untreated, OSA may cause impaired cognitive ability, road traffic accidents, cardiovascular morbidity and all-cause mortality [1].

Various therapeutic options exist. CPAP is the standard treatment for adult OSA, although its clinical application can be compromised by intolerance and poor compliance [2], while adenotonsillectomy is the primary treatment option for children with OSA and adenotonsillar hypertrophy [3].

The purpose of this Special Issue entitled “Obstructive Sleep Apnea (OSA)” is to contribute to a better understanding of this underdiagnosed and often underestimated medical condition by bringing to light recent developments. The collection contains ten articles in the form of seven original studies and three review articles.

Despite recent advances in our understanding of OSA, certain aspects of the disease’s pathophysiology have yet to be fully elucidated. The cyclic pattern of intermittent hypoxia in OSA triggers oxidative stress, contributing to cellular damage. The review by Lavalle et al. (Contribution 7) explored the relationship between OSA and oxidative stress, shedding light on the molecular mechanisms involved and some potential therapeutic interventions. 

If left untreated, OSA represents a significant mortality risk. Among other morbidities, OSA is considered to be a risk factor for erectile dysfunction. The study by Martynowicz et al. (Contribution 8) found that subjects with erectile dysfunction have altered sleep architecture, oxygen saturation parameters and increased daytime sleepiness.

An early and accurate diagnosis of OSA is of paramount importance and, although overnight polysomnography remains the gold-standard diagnostic tool, a detailed patient history is always valuable. Several questionnaires have been developed to assist in the process of screening patients with suspected OSA and one of the simplest and most frequently used is the Epworth Sleepiness Scale (ESS), which measures daytime somnolence. This parameter is often measured differently by patients and their partners and there is still confusion regarding the utility of partner-completed ESS in identifying OSA and predicting its severity. Chaidas et al. (Contribution 3) showed that there is a strong correlation between patient- and partner-reported ESS scores, but neither patient- nor partner-completed ESS were associated with OSA severity.

A thorough clinical examination of the upper airway is essential in patients with OSA as the findings can help in guiding appropriate treatment. This Special Issue presents some new suggestions regarding the assessment of patients with OSA. Specifically, Morato et al. (Contribution 1) suggested a new tool for palatopharyngeal muscle assessment during intraoral examination, which may be useful for creating a common language for sleep surgeons. Moreover, the use of artificial intelligence (AI) in medicine is becoming increasingly popular. A systematic review by Tsolakis et al. (Contribution 9) demonstrated that automatic airway segmentation is accurate, fast and easy to use in the measurement of airways. Thus, the future use of AI in assessing airway patency in OSA patients is promising at the very least. 

CPAP is the first-line treatment for adult OSA but its efficacy may be limited by the variability that is seen in the rates of compliance with such treatment. For that reason, precise treatment assessment is particularly important. Brajer-Luftmann et al. (Contribution 4) showed that the automatic algorithm that is used in auto-CPAP measurement is a good tool for the assessment of the treatment efficacy of CPAP in home settings. 

In addition to CPAP, various alternative therapeutic options for OSA exist in published guidelines worldwide, including lifestyle changes, oral appliances and surgery, with a remarkable variation in their availability across different countries [4,5].

Weight loss plays an important role in OSA management with the aim of at least improving its severity. Considering the fact that OSA and systemic inflammation typically coexist within a vicious cycle, the study by Georgoulis et al. (Contribution 5) explored the effectiveness of a weight-loss lifestyle intervention in reducing plasma tumor necrosis factor-alpha (TNF-a), a well-established modulator of systematic inflammation, and concluded that a healthy lifestyle intervention may reduce systemic inflammation in patients with OSA.

Furthermore, OSA is often associated with craniofacial and orthodontic abnormalities, especially in children, and may require a maxillofacial and/or other orthodontic intervention in selective cases. Caruso et al. (Contribution 2) evaluated the outcomes of orthodontic treatment with a rapid maxillary expander in association with a Delaire mask, demonstrating improvements in airway patency and OSA-related clinical conditions in children with a class III malocclusion.

Tonsillectomy and uvulopalatopharyngoplasty (UPPP) are common procedures that are used in the surgical management of OSA. A study by Hu et al. (Contribution 6) showed that combining a tonsillectomy and UPPP in patients with OSA did not increase the risk of patients developing a deep neck infection in the long term, but may reduce its severity by decreasing the intubation rate and the length of hospitalization.

Although adenotonsillar hypertrophy is the primary cause of OSA in children, the airway pathology in adults is usually more complex with the presence of a multi-level obstruction. The critical role of epiglottis in airway narrowing has recently been revealed. A systematic review by Vallianou and Chaidas (Contribution 10) evaluated surgical treatment options for epiglottic collapse, demonstrating that all of the currently available surgical techniques are safe and effective in managing selected patients. Effective management of epiglottic collapse can improve OSA severity or even cure OSA, but can also improve CPAP compliance. The selection of an appropriate surgical technique should be part of an individualized, patient-specific therapeutic approach. 

In summary, this Special Issue offers further insights into the pathophysiology, diagnostic assessment and management of patients with OSA, and highlights the importance of continuous research in this field.
